# Relation of dietary insulin index and dietary insulin load to metabolic syndrome depending on the lifestyle factors: Tehran lipid and glucose study

**DOI:** 10.1186/s13098-022-00968-w

**Published:** 2022-12-30

**Authors:** Bayyeneh Khoshnoudi-Rad, Somayeh Hosseinpour-Niazi, Maryam Javadi, Parvin Mirmiran, Fereidoun Azizi

**Affiliations:** 1 Children Growth Research Center, Research Institute for Prevention of Non-Communicable Diseases, Qazvin University of Medical Sciences, Bahonar Blvd, P.O. Box: 34185-754, Qazvin, Iran; 2 Nutrition and Endocrine Research Center, Research Institute for Endocrine Sciences, Shahid Beheshti University of Medical Sciences, No. 24, A’rabi St., Yeman Av., Velenjak, P.O. Box: 19395-4763, Tehran, Iran; 3 Department of Clinical Nutrition and Dietetics, Faculty of Nutrition Sciences and Food Technology, National Nutrition and Food Technology Research Institute, Shahid Beheshti University of Medical Sciences, Tehran, Iran; 4 Endocrine Research Center, Research Institute for Endocrine Sciences, Shahid Beheshti University of Medical Sciences, Tehran, Iran

**Keywords:** Dietary insulin index, Dietary insulin load, Metabolic syndrome, Lifestyle factor

## Abstract

**Aim:**

The hypothesis of the effect of the insulinogenic effects of diet on the development of cardiovascular diseases has been proposed, but the findings of previous studies are very contradictory. We investigated the association between dietary insulin index (DII) and dietary insulin load (DIL), and metabolic syndrome (MetS) risk. Another objective was to examine the extent to which lifestyle (physical activity, smoking status, and weight change) and sex influence the relationship between DII, DIL, and MetS risk.

**Materials and methods:**

We followed 1915 participants in the Tehran Lipid and Glucose Study. DIL and DII were calculated based on a validated food frequency questionnaire. Weight change was measured, and participants were categorized into > 3% weight loss, weight stable (± 3%), and > 3% weight gain. By joint classification, the association between DII and DIL (< median and ≥ median) and risk of MetS was assessed according to weight change, sex, physical activity levels, and smoking status. Cox proportional hazards models were used to estimate the HRs (95% CI), adjusting for potential confounders.

**Results:**

During 8.9 years of follow-up, among 1915 participants, we documented 591 new cases of MetS. DII and DIL were not associated with MetS risk in the crude and adjusted models. However, DIL and DII were associated with weight gain (≥ 3%). In the crude model, DIL and DII were associated with a higher risk of weight gain [HR: 1.74: 95% CI 1.50–2.03, and 1.70 (1.46–1.98), respectively]. These associations remained significant after further adjustment for confounders. The HRs were 1.61 (1.35–1.92) for DIL and 1.64 (1.39–1.93) for DII. Among men, women, participants with low physical activity levels, and smokers, the risk of MetS, independent of DIL and DII, only increased in a participant with weight gain. In women with weight stability, DIL and DII, higher than the median, were positively associated with MetS risk.

**Conclusion:**

Our findings suggest that the association between MetS risk and a hyperinsulinemic diet depended on weight change.

## Introduction

Metabolic syndrome (MetS) is a multifactorial metabolic disease characterized by hypertension, dysglycemia, dyslipidemia, and central obesity. During the last two decades, the epidemic of MetS has been increasing at an alarming rate [1–3]. Its prevalence is increasing by more than 40% in some regions, in both developed and developing countries [1, 2]. Hyperinsulinemia and subsequent insulin resistance were well known to link the diet and MetS [4, 5]. Growing evidence suggests long-term exposure to insulin concentrations increases the risk of chronic disease [6, 7]. Although carbohydrate evokes postprandial insulin secretion and leads to hyperinsulinemia, the quantity, and quality of other macronutrients, including fat and protein, induce insulin secretion and hyperinsulinemia [8–10]. Two new dietary indices, the dietary insulin index (DII) and dietary insulin load (DIL), have been developed to assess the effect of insulinotropic factors of diet on insulin secretion and insulin exposure [11, 12]. The hypothesis of the effect of the insulinogenic effects of diet on the development of cardiovascular diseases (CVD) has been proposed, but the findings of previous studies are very contradictory. In cross-sectional studies, an insulinogenic diet has shown conflicting results with MetS and its components, including dyslipidemia, dysglycemia, and C-reactive protein concentrations [13–16]. Moreover, whether long-term consumption of diet-induced insulin secretion is associated with chronic diseases such as CVD and type 2 diabetes mellitus remain controversial in the general population [17, 18]. This discrepancy may be due to the modification effect of other lifestyle factors, such as physical activity and smoking status, particularly changes in body weight, on the association between DIL and DII and chronic diseases [13, 19, 20]. According to previous studies, following the consumption of insulinogenic food during the long term can increase the risk of weight gain [19, 21]. Changes in body weight have been shown to be associated with the incidence of chronic disease [22]. In addition, the development of chronic diseases such as MetS was influenced by two lifestyle factors, including physical activity and smoking status [23] and the interaction between DII and DIL with physical activity and smoking status on MetS was not evaluated yet. Few studies investigated the modification effect of weight change, physical activity, and smoking status on the relation between the potential insulinemic effect of diet on weight gain [13, 16, 19]. Therefore, the present study had multiple objectives. In this population-based prospective study, the first was to investigate the association between DII and DIL, weight change, and MetS risk. Another aim was to examine the extent to which lifestyle (physical activity, smoking status, and weight change) and sex influence the relationship between DII, DIL, and MetS risk.

## Materials and methods

### Study population

This study has been conducted within the framework of the Tehran Lipid and Glucose Study (TLGS), which is an ongoing prospective population-based study to prevent non-communicable diseases. The layout and other information on TLGS were provided elsewhere [24].

The TLGS was initiated in March 1999. A multistage, stratified cluster random sampling technique was used to enroll > 15,000 participants ≥ 3 years from district 13 of Tehran. The population of this district is representative of the urban population of Tehran, the capital city of Iran. Since 1999, the participants of TLGS underwent assessments for anthropometric measures, medication use, medical history of CVD risk factors, lifestyle factors, sociodemographic factors, socioeconomic status, and biochemical and blood pressure measurements. This information was documented through face-to-face interviews with the local research team every 3 years. Up to now, 6 phases of the examinations have been performed. Phases II, III, IV, V, and VI were prospective follow-up studies conducted during 2002–2004, 2005–2008, 2008–2011, 2012–2015, and 2016–2018, respectively.

The current study used the baseline examination data from phase III of the TLGS (2006–2008) because of the small sample size for dietary assessment in phases I and II of the study and using 24-h recall. The subjects were followed up to phase VI of TLGS (2016–2018).

During phase III of the TLGS (2006–2008), medical history and physical examination were collected for 12,523 participants. Owing to the cost, complexity, and time involved in the collection of dietary data in a large population, a representative sample of 4920 participants was randomly selected based on their age and gender. Of 4920 participants, 3462 agreed to complete a food frequency questionnaire (FFQ). The characteristics of participants who completed the FFQ were similar to those of the total population in phase III of TLGS [25]. For the current study, of 3462 participants, 3265 adults aged 19 years or older with complete data (demographic, anthropometric, biochemical, and dietary data) were selected from phase III (2006–2008). Moreover, individuals with MetS at baseline (n = 879), women who were pregnant or lactating at baseline and during follow-up (n = 28), and subjects with under- or over-reporting of energy intakes (daily energy intake < 500 and > 4200 kcal per day) (n = 115), participants following any specific diet as a result of their hyperlipidemia, hypertension, and hyperglycemia (n = 26), and subjects with missing biochemical and anthropometric measures related to diagnosis of MetS during follow up (n = 309) were excluded from the study. Final analysis was conducted on 1915 participants until 2018, with a response rate of 66%, during the 8.9 (Interquartile range: 7.98–9.69) year follow-up. The study protocol was approved by the Ethics Committee of the Research Institute for Endocrine Sciences (RIES) of Shahid Beheshti University of Medical Sciences, Tehran, Iran. Written informed consent was obtained from all participants.

### Anthropometric measurements

Briefly, the participants’ weight, while being minimally clothed without shoes, was recorded using a digital scale (Seca 707; range: 0–150 kg; Seca GmbH, Germany) and recorded with accuracy of 100 g. Height was also measured in a standing position, without shoes, with shoulders in neutral alignment using a stadiometer (Seca 225; Seca GmbH, Germany) and recorded to the nearest 0.5 cm. Body mass index (BMI) was calculated as weight (kg) divided by the square of height (m^2^). Waist circumference (WC) was measured at the umbilical level using an un-stretched tape measure without any pressure to the body surface (accuracy, 0.5 cm).

### Assessment of other variables

After participants rested in a sitting position for 15 min, blood pressure was measured using a standardized mercury sphygmomanometer (calibrated by the Iranian Institute of Standards and Industrial Research) on the right arm twice, at least 30 s apart, and the average of the two measurements was reported as the participant’s blood pressure. Demographic, lifestyle (smoking status and physical activity), socioeconomic status (education and employment), medication regimen (e.g., antihypertensive, lipid-lowering, and anti-diabetes drugs), and medical history were gathered using a questionnaire.

Physical activity was assessed using a modifiable activity questionnaire (MAQ) that included a list of all three forms of activities, including leisure time, job, and household activities. The frequency and amount of time spent per week on physical activity over the last year were recorded [26]. The physical activity levels were expressed as metabolic-equivalent (MET) hours per week (MET-h/week) [27]. The reliability and validity of the Persian version of the MAQ have been reported [28].

### Dietary assessment

During face-to-face interviews with expert dietitians, a validated semi-quantitative FFQ was used to determine the frequency of daily, weekly, or monthly consumption of each food item during the previous year [29]. The Iranian food composition table (FCT) was used to calculate macro- and micronutrient intake [30].

From the initial number of 1915 participants at baseline, 592 participants completed all 4 FFQs (at baseline and during follow-up in phases IV, V, and VI), 804 participants completed 3 FFQs (at baseline and in two of the three phases of the follow-up study), 316 participants completed 2 FFQs (at baseline and in one of the three phases of the follow-up study), and 203 participants did not complete any FFQs during follow-up (only at baseline). To impute missing values, last observation carried forward method was used. In the present study, due to the crucial effect of recent dietary intakes on the association between diet and chronic disease, we used an alternative approach according to the Hu et al. formula [31]. This approach, which is more important than the baseline measures, adds more weight to the recent diet, reduces within-subject variability, and evaluates the long-term diet.

### Definition of DIL and DII

Food insulin index (FII) refers to the incremental insulin area under the curve over 2 h in response to the consumption of a 1000-kJ (239 kcal) portion of the test food divided by the area under the curve after the ingestion of a 1000-kJ (239 kcal) portion of the reference food. The insulin index for 68 food items was obtained from studies by Bao et al. [12] (50 items), Bell et al. [32] (13 items), and Holt et al. [11] (5 items). The insulin index for three food items, including tea, coffee, and salt, was considered 0 because these foods’ energy, carbohydrate, protein, and fat content were close to 0. For the remaining 49 food items that were not available in the food lists of the mentioned studies, the FII of similar food items was used based on the correlation between their energy, fiber, carbohydrate, protein, and fat content. For example, both dates and raisins are dried fruits. The energy, carbohydrate, fat, protein, and fiber content of both fruits are similar. Therefore, we used the insulin index of raisins for dates. The 120 items of the FFQ, the source of the FII, and the FII value are presented in DII and DIL in Relation to MetS: The Shahedieh Cohort Study (available at www.jandonline.org). To determine DIL, first, the insulin load of each food was calculated using the following formula: Insulin load of a given food = insulin index of that food × energy content per 1 g of that food amount consumed (g/d). By summing the insulin load of each food, DIL was obtained for each person. DII was then calculated for each participant by dividing DIL by total energy intake.

### Biochemical assessments

For biochemical measurements, after 12–14 h of overnight fasting, venous blood samples were collected in vacutainer tubes and centrifuged within 30–45 min of collection. The fasting plasma glucose (FPG), high-density lipoprotein-cholesterol (HDL-C), and triglyceride (TG) levels were measured in the TLGS research laboratory on the day of sample collection, using a Selectra 2 autoanalyzer (Vital Scientific, Spankeren, the Netherlands) and commercial kits (Pars Azmoon Inc., Tehran, Iran). FPG level was measured using an enzymatic colorimetric method with the glucose oxidase technique. The inter- and intra-assay coefficients of variation (CV) at baseline and after follow-up were both below 2.3%. TG was also assayed using an enzymatic colorimetric method with glycerol phosphate oxidase. HDL-C was measured after the precipitation of apolipoprotein B-containing lipoproteins with phosphotungstic acid. In baseline and follow-up assays, both intra- and inter-assay CVs were below 2.1% and 3.0% for TG and HDL-C. All samples were analyzed when the internal quality control met the acceptable criteria.

### Definition of MetS

According to the Joint Interim Statement, a MetS diagnosis requires the presence of three or more criteria [33], including (1) elevated glucose concentrations (FPG concentration ≥ 100 mg/dL) or treatment with anti-hyperglycemic medications; (2) elevated serum TG concentration (≥ 150 mg/dL) or treatment with anti-hypertriglyceridemia medications; (3) reduced serum HDL-C (< 50 mg/dL in women and < 40 mg/dL in men); (4) elevated blood pressure (≥ 130/85 mmHg) or treatment with anti-hypertensive medications; and (5) enlarged abdominal circumference (≥ 95 cm according to the population- and country-specific cut-off points for Iranian adults of both genders [34].

### Definition of weight change

Percentage weight change was calculated by subtracting the baseline weight from the follow-up one and multiplying it by 100. Participants were categorized as those who lost weight (≥ 3%), those with weight stability (± 3%), and those who gained weight (≥ 3%) [35].

### Statistical analysis

Data are reported as mean (SD) and median (25th and 75th percentiles) for continuous variables or percentages for categorical variables. DII and DIL were categorized into tertiles. Baseline characteristics and energy-adjusted dietary variables were described across the tertiles of DII and DIL, using the general linear model and Chi-square test for continuous and categorical variables, respectively. Moreover, Cox proportional-hazards regression models were used to estimate the hazard ratios (HRs) and their 95% confidence intervals (CIs) for the incidence of MetS and weight gain ≥ 3% across the tertiles of DII and DIL. The first model was crude, while the second model was adjusted for age, gender, smoking, physical activity, education levels, occupation status, total energy intake, and family history of diabetes, dietary fiber and dietary cholesterol (all variable that adjusted was at baseline). The third model was additionally adjusted for BMI at baseline. The linearity of trends was determined by integrating the median values of tertiles as continuous variables into the Cox regression models. Based on the multivariable Cox regression model, by joint classification, we estimated the HRs and 95% CIs for MetS, according to the weight changes, sex, physical activity levels and smoking status. All statistical analyses were performed in SPSS version 15.0 (SPSS Inc., Chicago, IL, USA), and *P*-values less than 0.05 were considered statistically significant.

## Result

During 8.9 years of follow-up, among 1915 participants, we documented 591 new cases of MetS. The mean (SD) age and BMI at baseline were 36.5 years (13.3) and 25.6 (4.5), respectively. 60% of the participants were men. The baseline characteristics of participants across tertiles of DII and DIL are presented in Table 1. Participants in the highest tertiles of DIL and DII were significantly younger, more likely to be smokers, less educated, and had weight gain. The intake of dietary variables of participants across tertiles of DII and DIL are presented in Table 2. Participants in the highest tertile of DII and DIL had a higher intake of energy, carbohydrate, total fiber, sugar-sweetened beverages, fruit, meats, processed meat, organ meat, whole grain, refined grain, nuts, and dairy products.

**Table 1:** Baseline characteristics of participants across tertiles of dietary insulin load and dietary insulin index

	Dietary insulin load			Dietary insulin index		
	T1	T3	P value	T1	T3	P value
Median intake (g/d)	30,531	129,542		14.7	55.8	
Range of intake (g/d)	≤ 47,449	≥ 92,720		≤ 21.4	≥ 39.6	
Age at baseline (y)	37.6 ± 0.5	36.2 ± 0.5	0.024	37.1 ± 0.5	36.8 ± 0.5	0.035
Female (%)	72.6	52.4	< 0.001	96.8	55.7	< 0.001
Physical activity (MET hour-week) at baseline	4.6 ± 0.3	4.9 ± 0.3	0.128	4.9 ± 0.3	4.8 ± 0.3	0.396
Smoker at baseline (%)	16.3	24.3	< 0.001	18.6	23.8	0.060
Academic degrees (%)	30.3	22.4	0.005	29.0	22.0	0.043
Occupational status, unemployed (%)	61.0	54.1	0.033	59.5	56.3	0.228
Family history of diabetes (%)	32.1	31.8	0.452	35.8	32.6	0.587
Family history of CVD events (%)	19.0	18.6	0.906	18.6	18.2	0.923
BMI at baseline (kg/m^2^)	25.8 ± 0.2	25.5 ± 0.2	0.359	25.8 ± 0.2	25.6 ± 0.2	0.200
Wight change (kg)	5.4 ± 1.2	11.0 ± 1.2	< 0.001	7.5 ± 1.2	10.8 ± 1.2	0.037

Values are mean ± SEM unless otherwise listed

*MET* metabolic equivalent, *BMI* body mass index

**Table 2:** Intake of dietary variables of participants across tertiles of dietary insulin load and dietary glycemic index

	Glycemic insulin load			glycemic insulin index		
	T1	T3	P value	T1	T3	P value
Total energy (kcal/d)	1993 ± 34	2705 ± 34	< 0.001	2183 ± 35	2399 ± 35	< 0.001
Carbohydrate (% of total energy)	62.3 ± 0.4	65.1 ± 0.4	0.002	60.0 ± 0.4	66.7 ± 0.4	0.016
Protein (% of total energy)	14.9 ± 0.3	14.4 ± 0.3	0.548	14.8 ± 0.3	14.4 ± 0.3	0.576
Fat (% of total energy)	30.8 ± 0.2	29.5 ± 0.2	< 0.001	30.9 ± 0.2	29.4 ± 0.2	< 0.001
SFA (% of total energy)	9.9 ± 0.1	9.8 ± 0.1	0.824	9.9 ± 0.1	9.8 ± 0.1	0.533
MUFA (% of total energy)	10.4 ± 0.1	10.2 ± 0.1	0.252	10.4 ± 0.1	10.1 ± 0.1	0.177
PUFA (% of total energy)	6.3 ± 0.1	6.2 ± 0.1	0.053	6.2 ± 0.1	6.1 ± 0.1	0.099
Total fiber (g/d)	39.1 ± 0.7	45.7 ± 0.7	< 0.001	43.0 ± 0.7	46.0 ± 0.7	0.001
Cholesterol (g/d)	202 ± 8	258 ± 8	< 0.001	224 ± 8	225 ± 8	0.085
Sugar sweetened beverages	24.7 ± 2.2	43.8 ± 2.2	< 0.001	28.9 ± 2.2	36.0 ± 2.2	0.036
Vegetables (g/d)	293 ± 6	288 ± 6	0.529	312 ± 6	262 ± 6	< 0.001
Fruit (g/d)	354 ± 11	423 ± 11	< 0.001	355 ± 11	387 ± 11	0.007
Meat, processed meat and organ meat (g/d)	23.8 ± 0.8	31.2 ± 0.8	< 0.001	26.4 ± 0.8	29.8 ± 0.8	< 0.001
Poultry and fish (g/d)	35.5 ± 9.0	59.0 ± 9.0	0.154	38.8 ± 9.0	52.4 ± 9.0	0.550
Whole grain (g/d)	113 ± 3	166 ± 3	< 0.001	127 ± 3	148 ± 3	< 0.001
Refined grain (g/day)	250 ± 6	419 ± 6	< 0.001	263 ± 6	395 ± 6	< 0.001
Nuts (g/d)	77.8 ± 2.7	90.2 ± 2.7	0.005	71.3 ± 2.7	85.5 ± 2.7	0.046
Legumes (g/d)	37.8 ± 1.1	34.6 ± 1.1	0.027	41.1 ± 1.1	29.9 ± 1.1	< 0.001
Dairy products (g/d)	327 ± 8	455 ± 8	< 0.001	361 ± 9	408 ± 5	< 0.001

Organ meat including liver, heart, and kidney

Values are mean ± SEM

*SFA* saturated fatty acid, *MUFA* mono unsaturated fatty acid, *PUFA* poly unsaturated fatty acid

Table 3 presents multivariable-adjusted hazard ratio (95% confidence interval) for MetS and weight gain across tertiles of DII and DIL. DII and DIL were not associated with MetS risk in the crude and adjusted models. However, DIL and DII were associated with weight gain (≥ 3%). In the crude model, DIL and DII were associated with a higher risk of MetS (HR: 1.74: 95% CI 1.50–2.03, and 1.70 (1.46–1.98), respectively). These associations remained significant after further adjustment for confounders. The HRs were 1.61 (1.35–1.92) for DIL and 1.64 (1.39–1.93) for DII.

**Table 3:** Multivariable adjusted hazard ratio (95% confidence interval) for metabolic syndrome and weight gain across tertiles of dietary insulin load and dietary insulin index

Variable	Tertiles of intakes			
	T1	T2	T3	P_trend_
Metabolic syndrome				
DIL				
Model 1	1	1.07 (0.87–1.29)	1.17 (0.96–1.43)	0.270
Model 2	1	1.04 (0.85–1.28)	1.03 (0.82–1.29)	0.911
Model 3	1	1.03 (0.83–1.26)	0.98 (0.78–1.24)	0.926
DII				
Model 1	1	0.94 (0.77–1.14)	1.18 (0.97–1.43)	0.072
Model 2	1	0.89 (0.73–1.10)	1.02 (0.82–1.26)	0.419
Model 3	1	0.92 (0.75–1.13)	1.01 (0.82–1.25)	0.625
Weight gain (≥ 3%)				
DIL				
Model 1	1	1.31 (1.13–1.53)	1.74 (1.50–2.03)	< 0.001
Model 2	1	1.19 (1.02–1.40)	1.58 (1.32–1.88)	< 0.001
Model 3	1	1.24 (1.06–1.46)	1.61 (1.35–1.92)	< 0.001
DII				
Model 1	1	1.19 (1.02–1.38)	1.70 (1.46–1.98)	< 0.001
Model 2	1	1.06 (0.90–1.24)	1.57 (1.33–1.85)	< 0.001
Model 3	1	1.13 (0.96–1.32)	1.64 (1.39–1.93)	< 0.001

Model 1 was crude

Model 2 was adjusted for age, gender, smoking, physical activity, education levels, occupation status, total energy intake, and family history of diabetes, dietary fiber and dietary cholesterol (all variable that adjusted was at baseline)

Model 3 was additionally adjusted for BMI at baseline

**Fig. 1:**
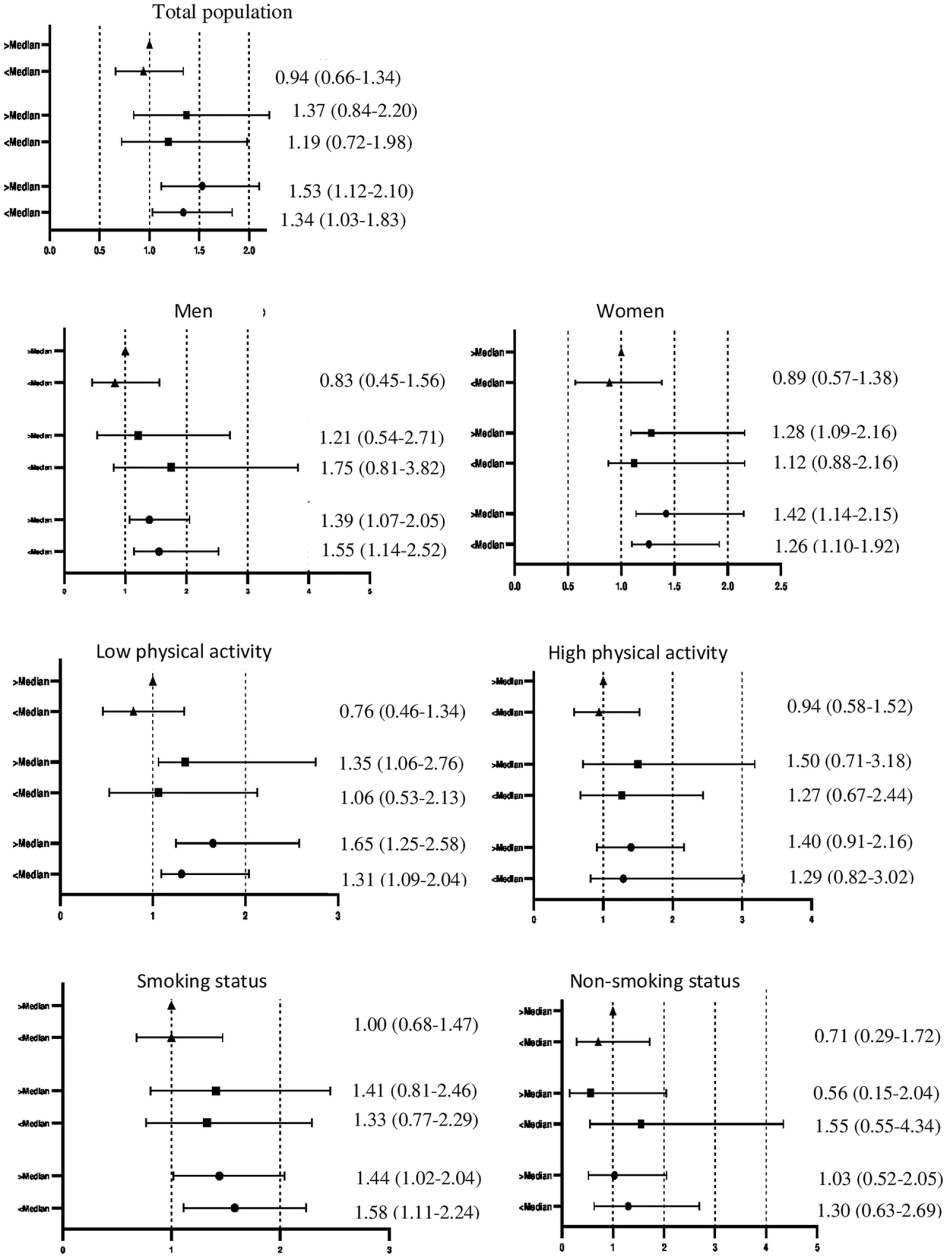
Hazard ratios of the combined. effect of dietary insulin load consumption (< median vs. ≥ median) and weight change (triangle, > 3% weight loss; square, weight stable (± 3%); and circle, > 3% weight gain) on risk MetS after adjustment for age, gender, smoking, physical activity, education levels, occupation status, total energy intake, and family history of diabetes, dietary fiber and dietary cholesterol and BMI at baseline

**Fig. 2:**
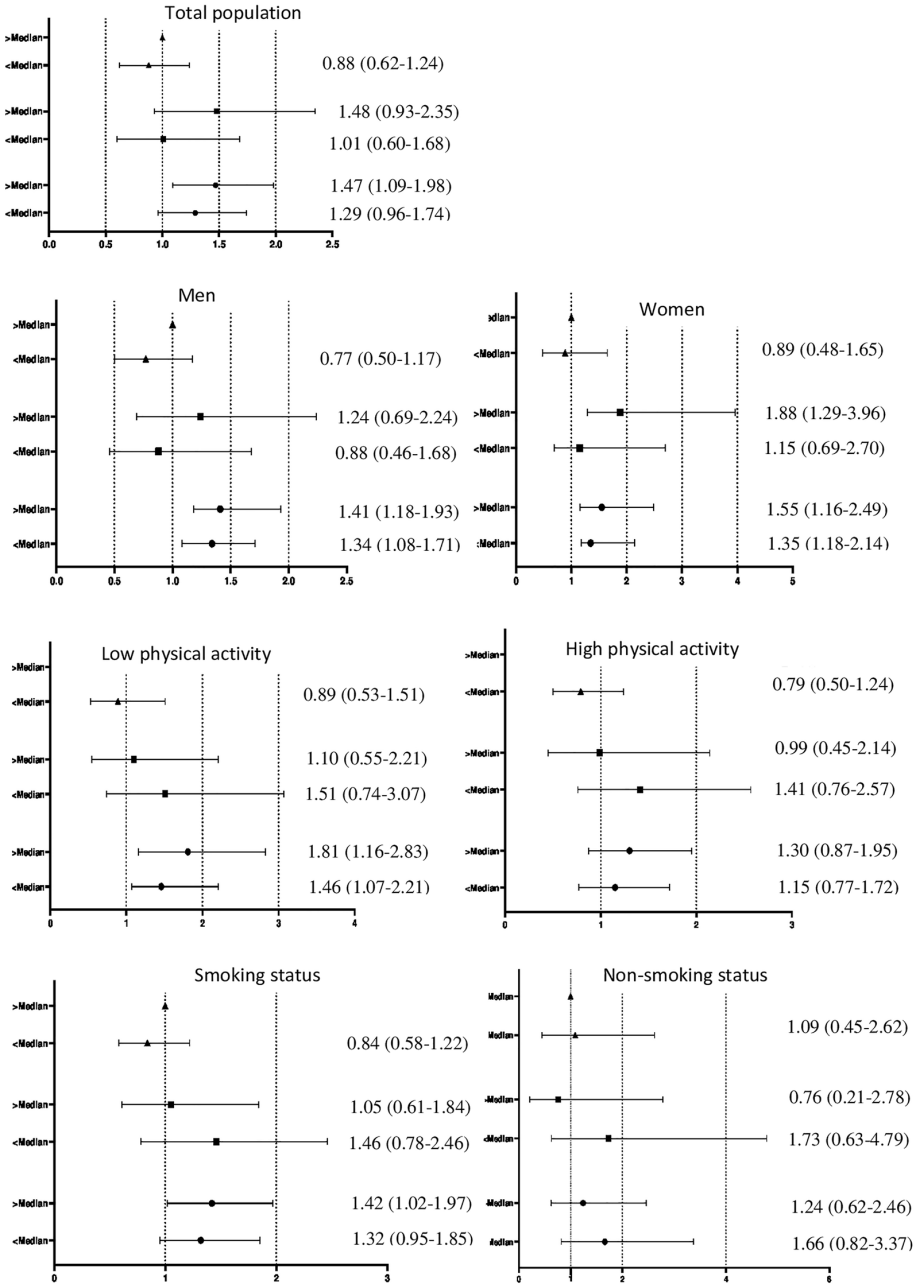
Hazard ratios of the combined. effect of dietary insulin index consumption (< median vs. ≥ median) and weight change (triangle, > 3% weight loss; square, weight stable (± 3%); and circle, > 3% weight gain) on risk MetS after adjustment for age, gender, smoking, physical activity, education levels, occupation status, total energy intake, and family history of diabetes, dietary fiber and dietary cholesterol and BMI at baseline

 The combined effects of weight change and DIL and DII on MetS risk in the total population, as well as by gender, physical activity level, and smoking status, are shown in Figs. 1 and 2. In the total population, the risk of MetS, independent of DIL, increased in a participant with weight gain. However, participants with weight gain and DII ≥ median exhibited a significantly higher risk of MetS than the reference group. This association was not observed among subjects with DII lower than the median. In participants with weight stability and weight loss, DIL and DII, lower or higher than the median, were not associated with MetS risk compared to the reference group.

Further analyses on sex stratification revealed an association between weight gain and risk of MetS in men, independent of DIL and DII. The risk of MetS was not associated with DIL among men with weight stable and weight loss categories. Among women, the risk of MetS, independent of DIL and DII, increased with weight gain. In women with weight stability, DIL and DII, higher than the median, were positively associated with MetS risk. This association was not observed among subjects with DIL, and DII lower than the median.

Stratification based on physical activity level resulted in the increase of MetS risk among weight gained group with low physical activity, independent of DII and DIL. No association was found between DIL, DII, and risk of MetS in both weight-stable and weight-loss participants. Among participants in the medium and high category of PAL, DII and DIL were not associated with MetS, regardless of weight change, after adjustments for confounding factors.

Among smoking participants with weight gain, DIL > or < of the median increased risk of MetS compared to participants with weight loss. In these participants, DII > of the median increased risk of MetS compared to participants with weight loss. This association was not observed among subjects with DII lower of the median. The risk of MetS was not associated in a participant with weight stability and weight loss across DIL and DII lower or higher than the median intake. In non-smoking participants, DIL and DII were not associated with MetS, regardless of weight change, after adjustments for confounding factors.

## Discussion

In this population-based prospective study, we found that DII and DIL were associated with weight gain but not the MetS. These associations survived after accounting for several confounders, including age, gender, and energy intake. After stratification of participants by weight changes, DII, lower or higher than the median intake, was positively associated with risk of MetS in the weight gain ≥ 3%, but not in the weight gain stable and loss weight groups. We also observed a higher risk of MetS with a diet high in DII and DIL, weight gain ≥ 3% and low physical activity level. Although weight gain, independent of DIL, increased the risk of MetS in both men and women, but in weight stable status, a higher dietary DII and DIL increased the risk of MetS in women.

Previous studies have investigated the association between DII and DIL, cardiometabolic risk factors, and chronic diseases, including MetS, type 2 diabetes mellitus, and CVD [3, 13, 14, 16–18, 36], but the conclusion of these studies has been controversial. This may be due to that BMI and insulin resistance status appeared to modify the association between DII, DIL, and metabolic diseases [13, 16, 17, 19, 36]. Sensitivity analysis based on dysglycemia [17], weight changes [19], and overweight/obese status [13] showed that the DII and DIL are related to cardiovascular risk factors and chronic diseases only in obese or overweight participants [13, 17, 20]. In cross-sectional studies, DII was significantly inversely associated with HDL-C, positively associated with high TG and unhealthy metabolic status in obese, but not in overweight or normal weight participants [13, 20]. In addition, DII and DIL were positively associated with the risk of CVD only among participants with dysglycemia [17]. Moreover, DII and DIL were positively associated with MetS and obesity risk among type 2 diabetes mellitus [36], but not in participants without diabetes [16]. In the current study, we also found a positive association between DII and DIL, and MetS existed in subjects with weight gain ≥ 3%. According carbohydrate-insulin model of obesity, a diet that induces insulin secretion through the anabolic effects of insulin cause weight gain and insulin resistance. In addition, an insulinogenic diet through decreasing satiety, increasing the total energy intake, and increasing insulin growth factor-1 (IFG-1) induce body fat accumulation and obesity [37, 38]. Following obesity and insulin resistance, insulin concentrations increase by reducing the clearance rate. In this condition, diets that stimulate insulin secretion to lead to long-term insulin exposure and the development of metabolic diseases such as type 2 diabetes mellitus and prediabetes [39]. In line with this evidence, in the current study, the positive association between DII and DIL, and MetS was observed only among subjects with weight gain ≥ 3%. These associations between DIL and MetS were stronger among participants with DIL ≥ median intake. These findings suggest that a high-insulinogenic diet, by causing obesity, leads to metabolic diseases such as type 2 diabetes mellitus, MetS, and CVD.

This study found no association between DII, DIL, and MetS in participants with weight loss. It seems that weight loss via decreased incidence of insulin resistance and insulin resistance remission [40], even with consumption of an insulinogenic diet, decreases circulating insulin levels by increasing the clearance rate. Following the low concentration of circulation insulin, the progress of metabolic diseases such as MetS can prevent.

In stratified analyses of DII and DIL and risk of MetS by weight change and physical activity level, we found that weight gain, independent of DIL and DII, low or higher than the median intake, increased the risk of MetS in participants with low physical activity levels. However, this association was stronger among participants with DIL ≥ median. In participants with medium/high physical activity levels, DII and DIL, lower or higher than the median intake, and weight gains > 3%, were not associated with the risk of MetS. In other words, if all adults have moderate/high physical activity levels, weight gain ≥ 3% and an insulinogenic diet would not be associated with the risk of MetS. Our findings are in line with a previous study that showed the associations between an insulinemic diet and 4-y weight gain were more substantial among participants who were less physical activity [19], and adjustment for physical activity disappeared the association between DII and DIL and health status [20]. In active participants, compared to sedentary individuals, low insulinemic diets were associated with a lower concentration of urinary and plasma C-peptide as a valid measurement of insulin resistance and insulin secretion [41, 42]. Previous studies reported that physical activity mediated the acute insulin response through improvement in insulin resistance [43], and in a participant with insulin resistance and type 2 diabetes mellitus, physical activity decreased insulin secretion and increased insulin clearance [44]. Physical activity is a key modifiable for reducing metabolic disease. To delay the onset of MetS, along with recommendations for consumption of a low insulinogenic diet and weight management, moderate/vigorous physical activity is also advised.

Recent meta-analyses of prospective studies found that sex modified the effects of dietary glycemic index and glycemic load on CVD outcomes [45–47]. In addition, high glycemic diets have shown an adverse effect on HDL-C and triglyceride concentrations in women compared to men [48]. Regarding the insulinogenic diet, cross-sectional studies have also reported that adherence to a diet with higher DII and DIL was positively associated with MetS and obesity only among women but not men [15, 21]. In the current study, we found that weight gain, independent of DIL, increased the risk of MetS in both men and women. But in the condition of weight stability, low DII and DIL diet in women appears to have a more beneficial effect than in men. Further research is warranted to assess the effect of modification of weight change on metabolic disease according to sex.

Cigarette smoking is associated with greater insulin concentration, hyperinsulinemia, insulin resistance, and MetS [49, 50]. In the current study, smokers who manage their weight had no risk of MetS, regardless of their diet. However, among smokers with a weight gain > 3%, the risk of MetS increased with the consumption of an insulinogenic diet. In contrast to the findings of our study, the association between Dietary Index for Hyperinsulinemia and weight gain is stronger among never-smoker participants [19]. The synergic effects of smoking and diet on the incidence of MetS and the effect modifier of weight change on this relationship need further investigation.

The strength of the current study was the population-based prospective design, use of validated FFQ, using alternative approaches for the assessment of dietary intake, and investigation of the mediatory effect of lifestyle factors on the association between DII and DIL and MetS risk. Nevertheless, the present study had some limitations. First and foremost, consuming foods together can affect the insulinemic response. However, assessment of dietary intake via FFQs cannot determine which foods are consumed together. The study was carried out in the metropolitan area of Tehran, which may limit the generalizability of our findings, especially in rural zones. Third, relatively low duration of follow-up (8.9 years) is another limitation of this study. Further prospective studies with a long follow-up period (≥ 20 year) are needed to better understand the association between DII and DIL and risk of MetS. Fourth, Lack of data on post load insulin and glucose concentrations to calculate insulin secretion indexes is another limitation of the current study. Additionally, even though data were adjusted for all confounding factors, residual or unmeasured confounding factors such as genetic factors that influence weight change in subjects with metabolic syndrome may also contribute to these association.

## Conclusion

Our findings suggest that the association between MetS risk and a hyperinsulinemic diet depended on weight changes. Moreover, the combined effects of weight changes and insulinogenic diet on the incidence of MetS were different among men and women. Furthermore, medium/high physical activity levels may offset the detrimental effect of weight gain and insulinogenic diet on MetS risk in weight gain status.

## Data Availability

The datasets generated and/or analysed during the current study are not publicly available due institution’s policy but are available from the corresponding author on reasonable request.

## Abbreviations

MetS: Metabolic syndrome
DII: Dietary insulin index
DIL: Dietary insulin load
CVD: Cardiovascular diseases
TLGS: Tehran Lipid and Glucose Study
FFQ: Food frequency questionnaire
RIES: Research Institute for Endocrine Sciences
BMI: Body mass index
WC: Waist circumference
Met: Metabolic-equivalent
MAQ: Modifiable activity questionnaire
FCT: Food composition table
FII: Food insulin index
FPG: Fasting plasma glucose
HDL-C: High-density lipoprotein-cholesterol
TG: Triglyceride
CV: Coefficients of variation
IFG-1: Insulin growth factor-1
